# Automatic Seizure Classification Based on Domain-Invariant Deep Representation of EEG

**DOI:** 10.3389/fnins.2021.760987

**Published:** 2021-10-15

**Authors:** Xincheng Cao, Bin Yao, Binqiang Chen, Weifang Sun, Guowei Tan

**Affiliations:** ^1^School of Aerospace Engineering, Xiamen University, Xiamen, China; ^2^Shenzhen Research Institute of Xiamen University, Shenzhen, China; ^3^College of Mechanical and Electrical Engineering, Wenzhou University, Wenzhou, China; ^4^Xiamen Key Laboratory of Brain Center, Department of Neurosurgery, The First Affiliated Hospital of Xiamen University, Xiamen, China

**Keywords:** electroencephalography, seizure classification, deep learning, domain-invariant representation, hybrid deep model

## Abstract

Accurate identification of the type of seizure is very important for the treatment plan and drug prescription of epileptic patients. Artificial intelligence has shown considerable potential in the fields of automated EEG analysis and seizure classification. However, the highly personalized representation of epileptic seizures in EEG has led to many research results that are not satisfactory in clinical applications. In order to improve the clinical adaptability of the algorithm, this paper proposes an adversarial learning-driven domain-invariant deep feature representation method, which enables the hybrid deep networks (HDN) to reliably identify seizure types. In the train phase, we first use the labeled multi-lead EEG short samples to train squeeze-and-excitation networks (SENet) to extract short-term features, and then use the compressed samples to train the long short-term memory networks (LSTM) to extract long-time features and construct a classifier. In the inference phase, we first adjust the feature mapping of LSTM through the adversarial learning between LSTM and clustering subnet so that the EEG of the target patient and the EEG in the database obey the same distribution in the deep feature space. Finally, we use the adjusted classifier to identify the type of seizure. Experiments were carried out based on the TUH EEG Seizure Corpus and CHB-MIT seizure database. The experimental results show that the proposed domain adaptive deep feature representation improves the classification accuracy of the hybrid deep model in the target set by 5%. It is of great significance for the clinical application of EEG automatic analysis equipment.

## Introduction

According to the World Health Organization (WHO), nearly 50 million people suffer from epilepsy worldwide, and it is estimated that 2.4 million people are diagnosed with epilepsy annually (World Health Organization, 2019). In order to start anti-epileptic drugs or treatment, timely and accurate diagnosis of epilepsy is very important. Nowadays, electroencephalogram (EEG) plays a significant role in epilepsy diagnosis. EEG is the record of electrical activity collected through electrodes on the scalp to provide spatial and temporal information of the brain. The neurologist checks the EEG for abnormal brain electrical activity, identify the type of seizure, and perform pathological analysis. However, manual analysis of EEG records is very time-consuming, especially with the increase of outpatient routine EEG and inpatient long term monitoring, and neurologists often spend several hours a day to review EEG (Krumholz et al., 2007). Therefore, the intelligent EEG analysis and classification algorithm has become the key to improving the level of epilepsy treatment.

The automatic analysis process of EEG can be roughly divided into several steps such as signal acquisition, preprocessing, feature extraction, and pattern recognition. Due to random and complex neural activity, EEG signals are inherently non-linear, non-stationary, and highly random. The filtering and noise reduction of the original EEG signal is very important for artifact recognition and feature extraction (Chen et al., 2019). According to clinical experience, epilepsy can cause changes in the waveform and frequency of EEG. Therefore, time-frequency analysis tools such as wavelet transform are widely used in EEG preprocessing and feature engineering. Mahjoub adapted tunable-Q wavelet transform and multivariate empirical mode decomposition to the preprocessing and feature extraction of EEG (Mahjoub et al., 2020). Slimen removed artifact by Savitzky–Golay filter and multi-scale principal component analysis (Slimen et al., 2020). He proposed a periodic overlapping group sparsity method for sparse feature extraction (He et al., 2018). Hussain proposed a novel entropy index permutation fuzzy entropy that significantly improves the accuracy of a variety of machine learning algorithms (Hussain et al., 2019). Gabara uses the CHB-MIT database to study the machine learning algorithm for identifying EEG during seizures. The experimental results suggest that indicators such as Fractal Dimension, Fluctuation Index, Variation Coefficient, and Kurtosis are more effective (Gabara et al., 2020).

Feature engineering reveals the difference between epileptic EEG and normal EEG, and also reduces the dimensionality of the data, so that machine learning models can be used to classify samples. Since the database disclosed in the early years only had two types of labels, normal and seizure, support vector machines (SVM), an algorithm particularly suitable for two classification problems, have been widely used. RM utilized SVM to identify seizure EEG signal (Aileni et al., 2019). Li proposed a novel EEG feature matrix generation algorithm based on fast Fourier transform, then used principal component analysis network for dimensionality reduction, and finally used SVM for identification, which were implemented on the Bonn database and the CHB-MIT database. The accuracy of 99 and 98% are achieved (Li and Chen, 2021). He proposed a diagnosis method based on sparse demodulation operator (He et al., 2019). Subasi used particle swarm optimization to determine the optimum parameters of SVM and increased the accuracy of epileptic seizure to 99.38% (Subasi et al., 2019). Experiments conducted by Shabarinath show that the combination of feature extraction *via* discrete wavelet transform and seizure identification *via* extreme learning machine achieved the highest accuracy, reported to be 90.1% (Shabarinath et al., 2019).

Despite many research cased combing feature engineering and machine learning have achieved high recognition accuracy on public databases. The modeling capabilities of machine learning models are limited, and severe degradation has occurred on large databases (Roy et al., 2019). The exciting thing is that, owing to the accumulation of database, deep learning models that can process big data through self-learning have been proven to be similar to humans in biomedical data analysis (Rajpurkar et al., 2017; Yıldırım et al., 2018; Arsenovic et al., 2020). Ozal uses a one-dimensional convolutional neural network (CNN) to analyze single-channel digital records to identify abnormal EEG, and achieves an accuracy of 79% on the Temple University Hospital EEG Abnormal Corpus (Yildirim et al., 2020). Liu applied smoothing and collar technique to the outputs of CNN and increased the sensitivity of seizure detection to 97% (Liu et al., 2019). Wang calculated the sub-band mean amplitude spectrum map (MAS) of the multi-channel EEG as a two-dimensional representation of the EEG and then trained the CNN to recognize the types of EEG samples (preictal, ictal, interictal), and the overall accuracy reached 92.77% (Wang Y. et al., 2019). Yang uses a convolutional autoencoder to extract features from EEG records and then uses a long short-term memory network (LSTM) to detect seizure (Li et al., 2020b). Abdelhameed uses multi-lead EEG as a monitoring method, uses a two-dimensional convolutional autoencoder to extract features from a time-channel two-dimensional matrix, and then uses LSTM to identify seizures. The accuracy achieved on the CHB-MIT database is reported above 98 (Abdelhameed and Bayoumi, 2021). Li uses the squeeze excitation module that can model the relationship between channels to adaptively select the EEG leads processed by the deep model. For the Temple University Hospital EEG Seizure Corpus (TUSZ), the accuracy of the proposed PSNE to identify epilepsy types is reported to reach 92% (Li et al., 2020a).

The series of deep models mentioned above achieve high accuracy on the database used. However, the manifestation of epilepsy in EEG is affected by various physiological indicators, and it is difficult to collect all possible databases. When the object monitored by the EEG classification model differs from the training data, the classification accuracy will inevitably decrease. The exciting thing is that deep learning experts provide a possible solution, which is transfer learning (Wang S.-H. et al., 2019; Cao et al., 2021; Zhuang et al., 2021). There have been studies exploring the application of transfer learning to ECG classification (Alghamdi et al., 2020; Naz et al., 2021). Wang proposed a continuous index adaptive algorithm, which extended the domain adaptive learning to the continuously changing domain (Wang et al., 2020). Owing to the powerful feature extraction of the deep model, deep domain adaptation has succeeded in machine vision, fault diagnosis, and other fields. Constraints were imposed on the deep representation, such as the maximum mean discrepancy (Cao et al., 2020), to align the distribution of the target domain samples and the source domain samples in the deep feature space, thereby improving the classification accuracy of the classifier in the target domain. Yin applies domain adaptation to personalized ECG monitoring combined with IR-UWB radar (Yin et al., 2019). Wang proposed an arrhythmia heartbeat classification method for ECG based on unsupervised domain adaptation.

In order to improve the reliability of EEG monitoring equipment in detecting seizure and classification in clinical applications, a hybrid deep network is proposed in this work, and a domain adaptive training method is proposed accordingly. First, we use the labeled samples in the database to train hybrid deep networks (HDN) to construct the deep feature map of EEG. Then, we use the unlabeled samples of the target patient to carry out domain adaptive learning, and fine-tune the HDN to improve the domain invariance of the deep re-representation. We realize the accurate classification of seizure.

## Methods

### Overview of the Proposed Method and Hybrid Deep Networks

**FIGURE 1:**
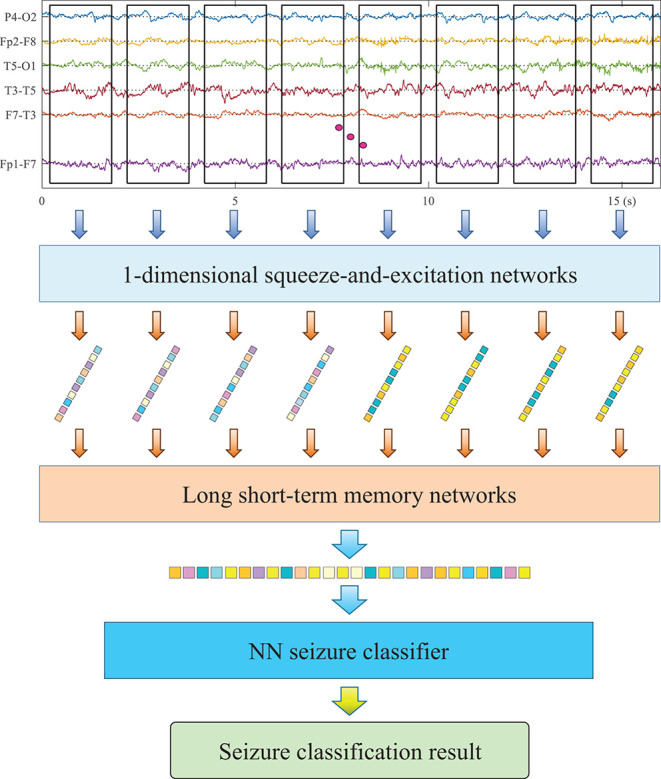
The architecture of the utilized hybrid deep networks (HDN).

The pipeline of the proposed method is illustrated in Figure 1, and the utilized HDN consists of three sub-networks, 1-D squeeze-and-excitation networks (SENet), LSTM, and Multilayer Perceptron (MLP). SENet and LSTM are responsible for extracting deep features of multiple time spans from EEG, and MLP is responsible for identifying seizure types based on the deep features of EEG. Firstly, the EEG monitoring device is used to collect the EEG of patients and then store it as a channel-time two-dimensional matrix. Taking the current moment as the origin, eight short-term EEG samples with a duration of 2 s are successively cropped to the previous. Firstly, we input the 2-s multi-lead EEG matrix into the 1-D CNN to extract deep features. The utilized 1-D CNN has as many input channels as EEG leads, and each input channel accepts one lead of EEG data. Then, the short-term deep feature vectors of the eight short-term EEG samples are sequentially input to the LSTM to extract long-term deep features. Finally, a classifier is used to identify the health status of EEG and predict the type of seizure.

### Short-Term Feature Extraction *via* Squeeze-and-Excitation Networks

According to the professional knowledge of neurology, epilepsy is manifested as changes in waveform and frequency in EEG. The superior performance of CNN in extracting waveform and frequency features has been widely verified in the field of machine vision and natural language processing. On the other hand, different types of seizure will cause changes in different leads, for example, local seizure only shows variation in several leads. Therefore, the deep network used should be able to model the relationship between channels and identify the differences between different leads. For this reason, this research adapted SENet to extract deep features from short-term EEG samples and reduce the dimensionality. The utilized SENet consists of five SE-Res blocks connected in series. The number of channels of each block is 20, 40, 80, 160, and 160. The length of the convolution kernel of each block is 16, 8, 5, 3, and 3.

The utilized SE-Res block constants consist of two convolutional layer, a global maximum pooling layer, an embedded fully connected neural network (NN), a scale block, a skip path, and an Add operation. The convolutional layer extracts feature from each channel and adjusts the number of channels. The global maximum pooling maps the feature maps to channel indices. The embedded NN takes the output of the global pooling layer as the input layer and then outputs the activation value of each channel. In order to simplify training and reduce calculations, the embedded NN uses only one hidden layer. The inventor of the SE module proposes to set the number of neurons in the hidden layer to one-sixteenth of the number of input channels. Considering the small number of channels in each module in this study, the number of neurons in the hidden layer is consistently set to 20. The first fully connected layer uses the *ReLU* activation function. The second fully connected layer uses the sigmoid activation function to map the activation value of the channel to the interval [0,1]. The scaling block assigns the activation value of the channel to each feature map. The structure of all SE-Res blocks in the model remains the same.

### Long-Term Feature Extraction *via* Long Short-Term Memory Network

The representation of seizure in EEG has strong individuality. It is difficult to balance the accuracy and generalization ability with a single short-term EEG sample to identify the seizure type of the patient. To this end, we use LSTM to extract the long-term features of EEG after SENet. Three LSTM modules are stacked to form a deep network, and after each LSTM module, the pooling layer is used for down-sampling. Inside the LSTM, a four-layer fully connected neural network is used to control each gate.

Suppose that *x^t−1^* and *x^t^* are the feature vectors extracted by SENet from the short-term EEG samples at time *t*−1 and time *t*, respectively. *h*^*t*−1^ is the output of LSTM with the input of *x*^*t*−1^, which is also called the hidden cell state. Three neural networks are used determine which historical information should be inherited into the processing of the feature vector at the current moment, which historical information should be updated, and extract features from the current input feature vector. The forget gate and input gate in LSTM are merged into an update gate *u^t^*, which determines which parts of the current information should be output and at the same time determines which historical information should be forgotten 1−*u^t^*. One sub-network is reduced, and calculation is easier. The output of LSTM and the operation of each gate in it are defined by the following equations:

(1) $$h^{t}=\left(1-u^{t}\right)\,⊙h^{t-1}\,u^{t}+u^{t}\,⊙{\tilde{h}}^{t}$$

(2) $${\tilde{h}}^{t}=t\,a\,n\,h\,\left(F_{o}\,\left(\left[h^{t-1}\,⊙r^{t},x^{t}\right]\right)\right)$$

(3) $$u^{t}=\sigma \,\left(F_{u}\,\left(\left[h^{t-1},x^{t}\right]\right)\right)$$

(4) $$r^{t}=\sigma \,\left(F_{r}\,\left(\left[h^{t-1},x^{t}\right]\right)\right)$$

Among them, ⊙ denotes pointwise multiplication and [⋅,⋅] denotes vector connection. *F*_*o*_(⋅),*F*_*u*_(⋅),*andF*_*r*_(⋅) denotes the sub-network of output, update gate, and reset gate, respectively. σ(⋅) denotes *sigmoid* activation function, and *tanh*(⋅) denotes *tanh* activation function. The *sigmoid* activation function maps the input to [0,1] to realize the function of the gate. The *tanh* activation function is used to adjust the range of alternative states to avoid gradient explosion.

### Training Strategy

The proposed HDN adopts a step-by-step training strategy. Firstly, the short-term feature extractor SENet is trained supervised. The SENet is divided into two parts, the last two fully connected layers form a short-term classifier *f*_*1*_, and the front-end part forms a short-term feature extractor *g_1_*. Using the labeled short-term EEG samples to train *g_1_* and *f_1_*, with cross-entropy loss function, the optimization target is as follows:

(5) $$\min_{\Theta_{g_{1}},\Theta_{f_{1}}}\frac{1}{N_{b}}\,\sum_{i=1}^{N_{b}}\sum_{c=1}^{M}y_{i,c}\,\text{log}\,\left({\hat{y}}_{i,c}\right)$$

Then, the long-term feature extractor LSTM is trained supervised. The short-term feature vector sequence output by *g_1_* is used as the input of LSTM. When training LSTM, the parameters of *g_1_* are fixed and no longer updated. Use *g_2_* to denote the parameters of the LSTM and *f_2_* to denote the parameters of the seizure classifier MLP. The cross-entropy loss function is also used. The optimization target is as follows:

(6) $$\min_{\Theta_{g_{2}},\Theta_{f_{2}}}\frac{1}{N_{b}}\,\sum_{i=1}^{N_{b}}\sum_{c=1}^{M}y_{i\,c}\,\text{log}\,\left({\hat{y}}_{i\,c}\right)$$

**FIGURE 2:**
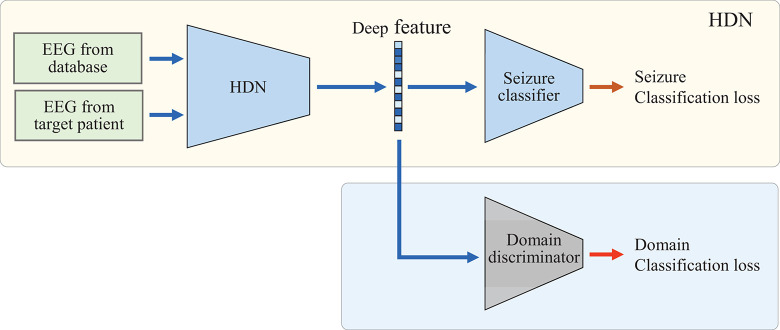
Domain adaptation of the proposed HDN based on adversarial learning.

Finally, the LSTM is fine-tuned *via* adversarial domain adaptation to realize domain-invariant deep representation. As shown in Figure 2, HDN, seizure classifier *f_2_*, and domain discriminator *f_d_* form an adversarial learning network. Among them, *f_2_* and *f_d_* both take the deep features output by HDN as input, and both are MLPs containing two hidden layers. In the domain adaptive learning phase, there are two inputs and two outputs. The model uses two datasets for train: one is the EEG dataset that has been labeled, and the other is the unlabeled EEG sample from target patient. The domain discriminator *f_d_* and HDN are optimized alternately. First, we freeze HDN and seizure classifier, and train *f_d_*. Because the domain index is known, the binary cross-entropy loss function is used. The optimization goal is shown as follows:

(7) $$\min_{\Theta_{f_{d}}}\frac{1}{N_{b}}\,\sum_{i=1}^{N_{b}}d_{i}\,\log\left({\hat{d}}_{i}\right)+\left(1-d_{i}\right)\,\log\left(1-{\hat{d}}_{i}\right)$$

After the domain classification loss converges, we freeze the parameters of *f_d_*, fine-tune the LSTM, and reduce the distribution discrepancy between the target domain and the source domain in the deep feature space. At the same time, the seizure classifier should be fine-tuned to maintain the classification accuracy in the source domain. Therefore, at this phase, the optimization target consists of two sub-items, as shown in Eq. 8.

(8) $$\min_{\Theta_{g_{2}},\Theta_{f_{2}}}\frac{1}{N_{b}}\,\sum_{i=1}^{N_{b}}\sum_{c=1}^{M}y_{i\,c}\,\text{log}\,\left({\hat{y}}_{i\,c}\right)-\frac{1}{N_{b}}\,\sum_{i=1}^{N_{b}}d_{i}\,\log\left({\hat{d}}_{i}\right)+\left(1-d_{i}\right)\,\log\left(1-{\hat{d}}_{i}\right)$$

The EEG classifier learns the spatial distribution of deep features under the supervision of labeled samples and optimizes the classifier. By minimizing the classification loss, the classifier can classify the labeled samples accurately. By minimizing the loss of deep distribution differences, the encoder can extract deep features from samples of different ages and different body mass index (BMI) that obey the same distribution. In this way, when EEG monitoring equipment is applied to people of different ages and BMI, it is not necessary to label the EEG of the target population manually. The unlabeled EEG can drive the model to adjust adaptively and realize the personalized arrhythmia monitoring based on EEG.

## Results

### EEG Database

The multi-class seizure type classification was implemented using the version 1.5.2 of Temple University Hospital (TUH) EEG Seizure Corpus (Roy et al., 2019). The train set of TUH EEG Seizure Corpus v1.5.2 collected 592 patient EEGs and recorded a total of 2,377 seizures. A total of eight types of seizure were recorded, of which three types had more than 50 seizures. The number of occurrences of other types is too less to train the deep model; they are not considered in this study. Therefore, in this study, the TUH database is used to carry out four classification experiments, which are Normal, Focal Non-Specific Seizure (FNSZ), Generalized Non-Specific Seizure (GNSZ), and Complex Partial Seizure (CPSZ).

The CHB-MIT database (Goldberger et al., 2000; Shoeb and Guttag, 2010) was also used in this research to verify the generalization of the proposed method when the test samples and training samples come from different hospitals and patients. The CHB-MIT database comes from 23 pediatric patients at Boston Children’s Hospital and an adult patient at Beth Israel Deaconess Medical Center. A total of 173 seizures judged by experts were recorded. The EEG is digitized at a sampling frequency of 256 Hz and divided into 1-h records for storage. After review by experts, the onset and end time of each seizure were recorded.

### Performance of Hybrid Deep Networks to Classify Seizure of EEG From Same Database

**FIGURE 3:**
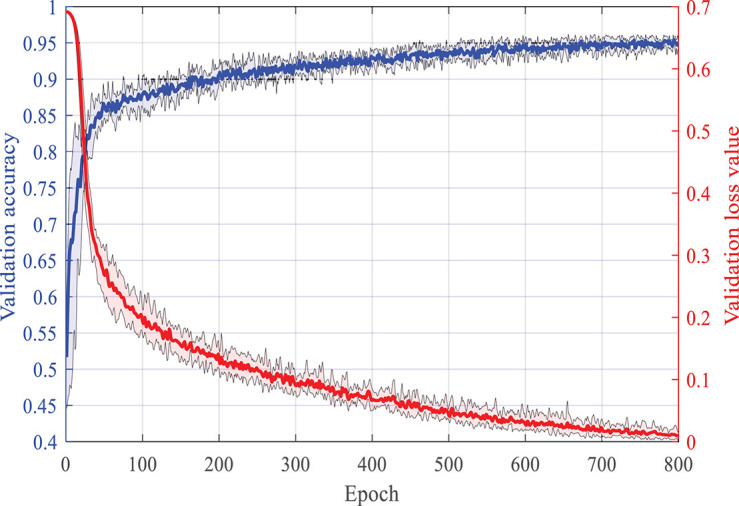
The learning process of HDN under the supervision of the expert label.

In this section, we use the TUH database to verify the classification accuracy of the proposed HDN after supervised training. We clip two samples from the EEG record of each seizure, one of which is centered on the midpoint of the seizure, and the other is centered on the starting point of the seizure. A total of 3,000 normal samples, 3,072 FNSZ samples, 818 GNSZ samples, and 566 CPSZ samples were clipped. The samples were randomly divided into six parts, of which six were used for training and one was used for testing, and a sixfold crossover experiment was performed. The Adam optimizer is utilized. The batch size is set to 128. The learning rate is set to 0.001. Figure 3 shows the change curve of accuracy and loss in the second training phase. The accuracy rate reaches 85% within 50 epochs and then rises slowly and steadily. At the 700th epoch, it reached 95% and stabilized. After the first phase of supervised learning, SENet achieved an accuracy rate of over 80%. Therefore, the accuracy of HDN can be increased quickly in the second training phase.

### Generalization of the Proposed Method to Classify Seizure for New Patients

In this section, we test the proposed method on different seizure classification tasks. We use different databases to train HDN and then test on different databases. The CHB-MIT database has only two types of labels, normal and seizure. Correspondingly, when using the TUH database to train the model, the samples are also relabeled as normal or seizure. Table 1 lists the test accuracy of using different methods to train HDN in each task. In each task, the training set is further divided into a training set and a validation set, and a sixfold crossover experiment is carried out to optimize the hyper-parameters and then tested on the test set. In order to comprehensively verify the superiority of the proposed method, we also trained the HDN using samples in the test set, and list the validation accuracy. Obviously, when the test set and training set are the same, HDN achieves the highest classification accuracy.

**TABLE 1:** The classification accuracy of hybrid deep networks (HDN) in the target domain trained by different method.

Train set	Test set	Trained with only train set	Trained with only test set	The proposed method
TUH train set	TUH dev set	92.4%	94.2%	94.7%
TUH dev set	TUH train set	71.2%	95.3%	80.3%
TUH train set	CHB-MIT set	89.7%	97.8%	96.3%
TUH dev set	CHB-MIT set	85.5%	97.8%	92.1%
CHB-MIT set	TUH dev set	80.3%	94.2%	86.7%

The fourth column of Table 1 lists the training accuracy achieved by HDN using each database for supervised learning. Among them, the CHB-MIT set is a normal/seizure two classification, and HDN achieves the highest training accuracy rate, reaching 97.8%. The training accuracy achieved in the TUH train set is the second, 95.3%. The training accuracy achieved in TUH dev set is the lowest at 94.2%. The author infers that this is because the same is a multi-classification task, but the TUH dev set samples are fewer.

Comparing the third and fourth columns, you can find the degradation of the trained HDN when applied to different test sets. The HDN trained with the TUH train set achieves a test accuracy of 92.4% in the TUH dev set, which is 2.9% lower than the training accuracy achieved by the HDN in the TUH train set, and is more accurate than the training achieved by the HDN in the TUH dev set. The rate dropped by 1.8%. This shows that under the condition of sufficient training samples, the recommended HDN has quite superior generalization ability. The high test accuracy of 89.7% achieved by the HDN trained with the TUH train set on the CHB-MIT set further validates this point. The HDN trained with the TUH dev set has the lowest test accuracy in the TUH train set, which is only 71.2%. Although HDN has achieved a training accuracy rate of over 94% in the TUH dev set, in the face of a larger and more complex TUH train set, the accuracy rate has dropped by more than 20%, losing its clinical application value. This verifies the importance of accumulating training data for automatic EEG analysis and diagnosis.

Finally, we compare column five and column three to verify the advantage of the proposed method. Following the proposed method, HDN first conducts supervised learning based on the training set. Then, we use the test set to carry out transfer learning to construct domain-invariant deep representation, thereby improving the adaptability of HDN in the test set. In task 1, the proposed method achieves a test accuracy of 94.7%, which exceeds the training accuracy achieved by HDN in the TUH dev set. When the training set is TUH dev set and the test set is TUH train set, the performance improvement of the proposed training method is the most significant, and the test accuracy rate is increased by 9.1%. When the test set and training set come from different patient groups, the proposed method increases the accuracy of HDN testing by more than 6 percentage points.

## Discussion

In this paper, we proposed a seizure classification method based domain-invariant deep representation of EEG. The proposed method is implemented by the HDN responsible for feature extraction and classification and the domain discriminator responsible for fine-tuning the deep representation. Benefiting from the advantages of the convolution kernel in extracting waveform features, after supervised learning, the CNN extracts deep features from the short-term EEG. The SE-Res module enables the modeling of the internal relationship of each lead, which improves the multi-classification capability of SENet. In the end, a seizure classification accuracy rate of 85.7 was achieved on the TUH database, which is a significant improvement compared to the machine learning model, but still cannot meet the needs of clinical applications. The author infers that this is because of the heterogeneity of seizure’s representation in EEG and the interference of complex and diverse non-stationary noise. The separability of short-term EEG samples in the deep feature space is insufficient, and it is difficult for a MLP to optimize an accurate classification hyperplane. CNNs can process EEG samples for a longer period of time, but require a huge amount of calculation, which conflicts with the limited computing power of monitoring equipment. Therefore, this article uses the trained SENet as a short-term deep feature extractor, and then uses an LSTM that is good at processing time series to extract long-term deep features. SENet, LSTM, and NN classifiers together form HDN. After stepwise supervised learning, a classification accuracy rate of more than 92% is finally achieved on the TUH database. The accuracy of detecting seizure on the CHB-MIT database reached 97.8%. The objective reasons include two points. One is that the CHB-MIT database contains far fewer patients than the TUH database, and the other is that the normal/seizure two-class classification task is more important and simple. However, under different combinations of training set and test set, the test accuracy of HDN has dropped by 3–13 percentage points compared to the evaluation accuracy. This shows that the generalization ability of HDN is still insufficient. What is exciting is that adversarial learning based on labeled data in the training set and unlabeled data in the test set can drive HDN to fine-tune the deep feature representation, so that the test set samples and training set samples are aligned in the deep space, thereby improving the test accuracy of the classifier. In task 2 where the test set is larger than the training set, the fine-tuning re-representation increased the test accuracy by 9.1 percent to 80.3%. When the test set and training set come from different hospitals and patient groups, the fine-tuning re-representation increased the test accuracy by more than 5 percentage points. In summary, the constructed HDN has strong EEG feature extraction capabilities, and the proposed adversarial learning method can adjust the deep feature mapping to achieve excellent domain-independent properties, which has important value for the clinical application of seizure automatic classification equipment.

## Data Availability Statement

The original contributions presented in the study are included in the article/supplementary material, further inquiries can be directed to the corresponding authors.

## Author Contributions

XC drafted the manuscript and performed all administrative tasks required for submission. BY took part in planning, supervision, and brainstorming the manuscript. BY and BC designed the classification method and contributed to the interpretation of results. WS contributed to the statistical analysis and interpretation of results. GT contributed to the identification of research topic and preparation of study design. All authors critically revised the manuscript and approved the final version.

## Conflict of Interest

The authors declare that the research was conducted in the absence of any commercial or financial relationships that could be construed as a potential conflict of interest.

## Publisher’s Note

All claims expressed in this article are solely those of the authors and do not necessarily represent those of their affiliated organizations, or those of the publisher, the editors and the reviewers. Any product that may be evaluated in this article, or claim that may be made by its manufacturer, is not guaranteed or endorsed by the publisher.
